# Analysis of contamination in cluster randomized trials of malaria interventions

**DOI:** 10.1186/s13063-021-05543-8

**Published:** 2021-09-10

**Authors:** Lea Multerer, Tracy R. Glass, Fiona Vanobberghen, Thomas Smith

**Affiliations:** 1 Swiss Tropical and Public Health Institute, Basel, Switzerland; 2 University of Basel, Basel, Switzerland

**Keywords:** Nonlinear analysis, Sigmoid random effects analysis, Malaria, Mosquitoes, Simulation study

## Abstract

**Background:**

In cluster randomized trials (CRTs) of interventions against malaria, mosquito movement between households ultimately leads to contamination between intervention and control arms, unless they are separated by wide buffer zones.

**Methods:**

This paper proposes a method for adjusting estimates of intervention effectiveness for contamination and for estimating a contamination range between intervention arms, the distance over which contamination measurably biases the estimate of effectiveness. A sigmoid function is fitted to malaria prevalence or incidence data as a function of the distance of households to the intervention boundary, stratified by intervention status and including a random effect for the clustering. The method is evaluated in a simulation study, corresponding to a range of rural settings with varying intervention effectiveness and contamination range, and applied to a CRT of insecticide treated nets in Ghana.

**Results:**

The simulations indicate that the method leads to approximately unbiased estimates of effectiveness. Precision decreases with increasing mosquito movement, but the contamination range is much smaller than the maximum distance traveled by mosquitoes. For the method to provide precise and approximately unbiased estimates, at least 50% of the households should be at distances greater than the estimated contamination range from the discordant intervention arm.

**Conclusions:**

A sigmoid approach provides an appropriate analysis for a CRT in the presence of contamination. Outcome data from boundary zones should not be discarded but used to provide estimates of the contamination range. This gives an alternative to “fried egg” designs, which use large clusters (increasing costs) and exclude buffer zones to avoid bias.

**Supplementary Information:**

The online version contains supplementary material available at (10.1186/s13063-021-05543-8).

## Background

Cluster randomized trials (CRTs) are often used in public health research to avoid contamination effects (also called indirect effects or spill-over effects) leading to averaging of estimates of effectiveness across the arms of a trial population in an individual-level randomized trial. The full effect of the intervention is only observed in comparisons of distinct clusters of individuals, but it may be difficult to ensure full separation between the intervention and control arms of the trial. This problem has long been recognized in the design of CRTs, especially for vaccine studies [1–4]. With directly transmitted diseases, dynamic models of the transmission across contact networks can provide an efficient, though technically challenging approach to optimizing trial design and estimating effects of contamination on effectiveness estimates [5].

With diseases transmitted by vectors, construction of contact networks is usually impossible and clusters are defined to correspond to the places where people get infected. In the case of *Aedes* transmitted diseases like dengue or zika, these may be schools or workplaces, since biting happens during the day. However, *Anopheles* mosquitoes transmitting malaria bite in the early night and early morning. Hence, most transmission of malaria is indoors or peri-domestic and can be geolocated to the host’s primary residence. In trials of interventions, such as the deployment of insecticides or distribution of bed nets, clusters are therefore defined as geographically congruent areas, with contamination effects mainly induced by mosquito movement because people living nearby might benefit from a reduced density of infectious mosquitoes. Other contamination effects that are unrelated to geographical distance, such as relocation of human hosts, are relatively unimportant. The maximum effect of intervention is then observed only where high coverage is achieved throughout a substantial group of neighboring individuals. Since *Anopheles* mosquitoes can fly several kilometers [6], trial arms need to be separated by large distances if contamination at cluster boundaries is to be avoided. This has led to CRTs with clusters of much larger geographical size than are required to estimate the effect of the intervention with wide buffer zones around each cluster where the intervention is introduced but excluded from data collection and analysis (a so-called fried egg design [7–12]).

With the fried egg design, a simple mixed effects model provides a valid analysis [7], providing the buffer zone is large enough. But because the intervention must be introduced in the buffer zone, the trial may be very expensive if there are high per capita intervention costs. Since the buffer zone is excluded from data collection, there are usually no data on whether the buffer is large enough to avoid contamination effects, and an unexpectedly large contamination leading to substantial bias in the estimate of effect would go undetected. These considerations challenge the rationale for fried egg designs. Recently, an alternative was proposed to a simple fried egg design by either fully including or excluding clusters from both the intervention assignment and the analysis based on a criterium of closeness between households to attain a better separation between intervention and control arms [13]. This approach leads to smaller trials and a conventional analysis can be carried out, but, depending on the proximity of clusters, is very computationally expensive and information on the contamination range is still needed to design such a trial.

There are reasons why contamination effects should be measured [13–15]. Evidence on contamination effects supports inference about indirect effects of the intervention, thus analyses of contamination in CRTs of insecticide-treated nets (ITNs) against malaria [16–18] fed into the rationale for massive distribution of the nets across Africa. In the largest trial in Asembo, Kenya [18], significant protective effects of ITNs were found for distances of up to 300 m from cluster boundaries, while on the coast effects persisted for distances of up to 1.5 km [17]. In these analyses, a linear model was extended to include a term of the distance to the nearest discordant observation. Nevertheless, it is not possible to obtain a closed-form range that specifies the maximal measurable extent of contamination from a linear model. Methods that jointly estimate contamination effects and adjust the estimate of effectiveness accordingly are needed. Neither the maximum distance that mosquitoes can fly, nor the distance over which contamination effects can be measured, necessarily equates with the distance over which contamination between trial arms is statistically relevant, and if contamination only biases the intervention effects over short distances then clusters could be smaller. This could lead to more cost-efficient, smaller trials while adding a new outcome measure to the analysis of CRTs.

This work proposes an approach for simultaneously estimating the intervention effectiveness and the contamination range, defined as the extent of measurable contamination across the intervention boundaries in CRTs of malaria interventions. Simulations of CRTs of malaria interventions targeting mosquito densities and measuring prevalence as the outcome, for example with a rapid diagnostic test (RDT), were used to assess the model performance. Simulated mosquito movement leads to correlations between households and hence to contamination between intervention arms. The degree of mosquito movement, intervention efficacy, numbers of clusters, households per cluster, and the pattern of spatial clustering in both the human and vector populations rates were varied. A reanalysis of a CRT for assessing the effects of ITNs on child mortality in the Kassena-Nankana district in northern Ghana (Navrongo trial) with the proposed method illustrates the findings [16, 19].

## Methods

### Simulation of CRTs with contaminated intervention effects

The simulations of cluster randomized trials entailed generating simulated human populations at the household level, assigning disease distribution in the absence of intervention and implementation of intervention effects as follows:

#### Human populations and disease distribution in the absence of intervention

To approximate patterns of heterogeneous human dispersion, simulated human populations living in *N* households on a domain of *η*×*η* km^2^ were generated via a (modified) Thomas cluster process [20] (Table 1). This algorithm generates a uniform Poisson point process of parent points with intensity *α*_1_, the cluster centers, and then replaces each parent point with a bivariate normally distributed cluster of offspring points, the households, with a mean number of points per cluster *α*_2_ and a standard deviation of random displacement of a point from its parent *α*_3_. This algorithm is implemented in the R-package spatstat [21] via the function rThomas.

**Table 1:** Summary of the parameters used to generate the data sets

		Levels	Values
Fixed parameters used to generate the data sets			
*N*	Number of households in trial		2500
*η*	Domain size		5 km
*α*_1_, *α*_2_, *α*_3_	Parameters for the Thomas process [21]		4, 50, 0.25 km
*ξ*, *π*	Index households and bandwidth for the KDE		200, 0.5 km
*ζ*_1_, *ζ*_2_	Scaling of *C*_*i*_’s		0.2, 0.6
Varying parameters used to generate the data sets			
*E*_*s*_	Efficacy	2	0.2, 0.4
*σ*	Standard deviation	5	0.042, 0.106, 0.170, 0.234, 0.298 km
$\theta = 1.64 \sqrt {2}\sigma $	⇒ Contamination range		0.10, 0.25, 0.40, 0.55, 0.70 km
*c*	Number of clusters		20, 25, 30, 35
*h*	Households per cluster		30, 40, 50, 60, 70
*c*&*h*	Only combinations with 0.6*N*<2*hc*≤*N* households	8	
	Seed, sampled out of [1,100 000] with seed(-1)	100	

**Fig. 1:**
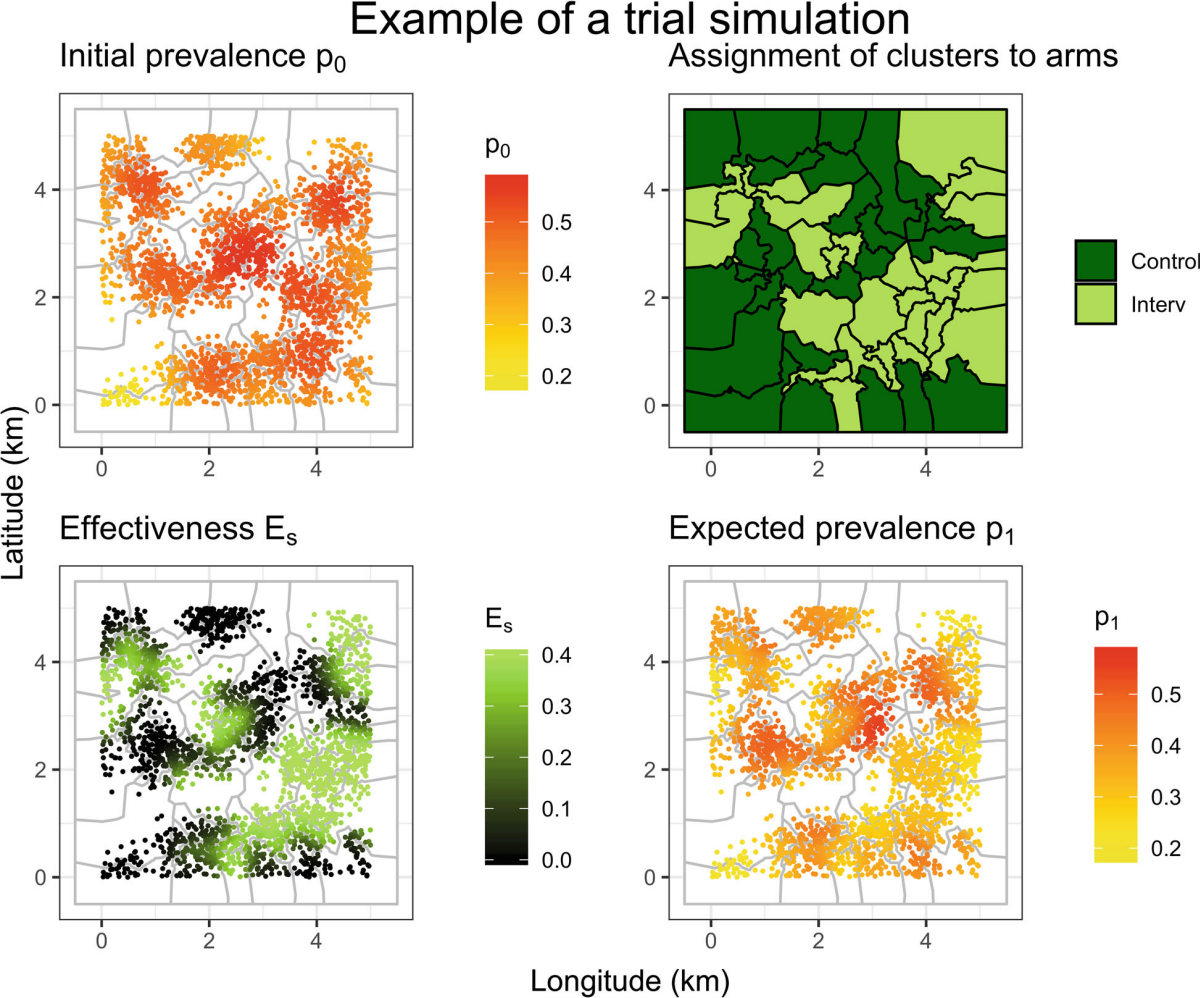
Trial simulation and intervention assignment, visualizing the assigned initial malaria prevalence to a distribution of households (upper left), the division of the households into clusters based on a travelling salesman algorithm together with the cluster assignment (upper right), the assigned effectiveness varying at the cluster boundaries due to the mosquito movement (lower left), and the resulting expected prevalence (lower right). The parameters used to generate this data set can be found in Table 1, with the varying parameters chosen as follows: households were assigned to 50 clusters, each consisting of 50 households. The intervention was assumed to be 40% effective and the assigned contamination range was 0.4 km

The simulated transmission potential was a smooth function in space with local maxima at a simple random sample of *ξ* index households. Each household *j* with coordinates *x*_*j*_ and *y*_*j*_ was assigned a local infection rate or vectorial capacity *C*_*j*_, represented as a function of its location (see upper left part of Fig. 1), generated as a sum of bivariate normal kernels centered on the index households with a bandwidth *π* and scaled to lie in [*ζ*_1_,*ζ*_2_].

Mosquito movement was simulated by further smoothing these infection rates *C*_*j*_ via a simple diffusion process [22]. The acquisition of infection from mosquitoes at each location is then proportional to *C*_*j*_. In the absence of intervention, these infections are distributed to other locations *i* proportionately to a bivariate normal kernel, where

$$\Sigma {\mathrel{\mathrel{\mathop:}=}} \left({\begin{array}{cc} \sigma^{2} & 0 \\ 0 & \sigma^{2} \end{array}} \right) $$

is the diagonal covariance matrix and *σ* is the standard deviation of the distance moved by mosquitoes during the extrinsic cycle of the parasite, i.e., the time it takes for a malaria parasite to become transmissible. Equivalently, the numbers of infections distributed to house *j* from house *i* is a Gaussian function of distance between the houses,

$$\begin{aligned} f_{i-j} &= f(x_{i}-x_{j},y_{i}-y_{j})\\ &{\mathrel{\mathrel{\mathop:}=}} \frac{1}{2\pi \sigma^{2}}\exp\left(\!-\frac{(x_{i}-x_{j})^{2}+(y_{i}-y_{j})^{2}}{2\sigma^{2}}\right) \!= f_{j-i}. \end{aligned} $$

This two-dimensional function results in a total dispersion of infections quantifiable by the trace of *Σ*, that is 2*σ*^2^. For each household *j*, this means that 95% of the dispersion of infections happens within a radius $\theta {\mathrel {\mathrel {\mathop :}=}} \Phi ^{-1}(0.95)\times \sqrt {2\sigma ^{2}}$ km where *Φ*^−1^(*p*) denotes the quantile function of a standard normal distribution with *p*∈[0,1]. Hereafter, *θ* is called the contamination range that quantifies the significant dispersion of infections (and hence the mosquito movement) in one direction.

The exposure to infection at location *j* in the absence of intervention is thus

$$z_{j,0} {\mathrel{\mathrel{\mathop:}=}} \sum_{i}\Big(C_{i} \frac{f_{i-j}}{\sum_{k}f_{j-k}}\Big), $$

where the normalizing term $\sum _{k}f_{j-k}$ is required to ensure that the total vectorial capacity distributed from household *j* over all destination houses sums to *C*_*j*_. The expected prevalence in household *j* in the absence of intervention, *p*_*j*,0_, is scaled so that the mean of *p*_*j*,0_ corresponds to a pre-defined value, $\bar {p}_{0}$. i.e.

$$p_{j,0} {\mathrel{\mathrel{\mathop:}=}} \bar{p}_{0}\frac{N}{\sum_{k} z_{k,0}} z_{j,0}. $$

#### Determination of clusters and assignment of intervention effects

The locations were grouped into *c* clusters per arm, each consisting of *h* households, by defining an efficient path through them with a heuristic algorithm for the traveling salesman problem (TSP) using the TSP package [23] in R, as proposed by Silkey et al. [24] for a trial of mosquito traps in Kenya [25], the SolarMal trial. Equal numbers of households were then allocated to each cluster along the derived path and a simple random sample of half the clusters was assigned to each arm of the trial (see upper right part of Fig. 1).

The presence of an intervention acting at source household *j* reduces the total number of infections acquired from mosquitoes in that household by some efficacy *E*_*s*_ so that *z*_*j*,1_, the exposure to infection of household *j* in the presence of the intervention, is

$$z_{j,1} {\mathrel{\mathrel{\mathop:}=}} \sum_{i}\Big(C_{i} \frac{f_{i-j}} {\sum_{k}f_{j-k}}(1-E_{s} \chi_{i})\Big), $$

where the indicator function *χ*_*i*_ takes the value 1 if household *i* is intervened, and the value 0 if it is in the control arm (see lower left part of Fig. 1). The expected prevalence in household *j* in the presence of intervention (using the same scale factor as for *p*_*j*,0_) is then

$$p_{j,1} {\mathrel{\mathrel{\mathop:}=}} \bar{p}_{0}\frac{N}{\sum_{k} z_{k,0}}z_{j,1}, $$

as shown in the lower right part of Fig. 1. For each household in the trial population, a single sample was drawn from a Bernoulli distribution with probability *p*_*j*,1_, such that

$$\kappa_{j} {\mathrel{\mathrel{\mathop:}=}} \left\{\begin{array}{ll} 1 &\text{with probability } p_{j,1},\\ 0 &\text{with probability } 1-p_{j,1}, \end{array}\right. $$

representing a malaria prevalence survey testing one person per household with an RDT for simplicity. This could easily be extended to more individuals per household by including another level of clustering in the Thomas cluster process.

#### Trial parameterization

The parameters of the simulation study were chosen to resemble a trial of mosquito traps in Kenya, the SolarMal trial [24, 25]. In this trial, clusters were assigned with a TSP and hence there were households within the contamination range spanning the cluster boundaries. In the upper part of Table 1, all fixed parameters of the trial simulation are listed. A domain of *η*×*η*, where *η*=5 km, was chosen. The three parameters required for the Thomas cluster process were chosen to be *α*_1_ = 4, *α*_2_ = 50 and *α*_3_=0.25 km, resulting in an expected number of 5000 households per realization. Of these households, *N*=2500 were chosen to represent the trial population (to have a constant trial population over different simulations). For the kernel density estimation (KDE) of a subsample of the households, *ξ*=200 households were randomly chosen with a bandwidth of *π*=0.5 km for the Gaussian kernel. The resulting pattern was then scaled to lay in between 0.2 and 0.6 for the initial prevalence.

In addition to the fixed parameters, four parameters of interest were varied, influenced by the values chosen or calculated for the SolarMal trial: the efficacy *E*_*s*_ (20% and 40%); the standard deviation of the Gaussian functions *σ*, resulting in a contamination range $\theta \,=\, 1.64 \sqrt {2\sigma ^{2}}$ km (*θ* = 0.1,0.25,0.4,0.55,0.7 km); five levels of cluster size for *h* (30, 40, 50, 60, 70 households per cluster); four levels of *c* (20, 25, 30, 35 clusters per arm), and of these 20 configurations of *h* and *c* only the ones with 0.6*N* < 2*hc* ≤ *N* were included to keep the number of observations stable (8 levels, (*c*,*h*) = (20,40), (20,50), (20,60), (25,40), (25,50), (30,30), (30,40), (35,30)). The theoretical intra-cluster correlation coefficient (ICC), a measure of variation of the outcome within clusters that is usually obtained from previous studies, was calculated for each data set, resulting in a mean ICC of 0.0021. This leads to an adequately powered study for an efficacy of 20% and an overpowered study for an efficacy of 40%, based on sample size calculations for malaria prevalence [7, 26, 27]. One hundred replicate data sets were produced using different seeds (and hence different patterns of households and infections) for each of the 2×5×8 parameter configurations. Following guidelines on simulation studies [28, 29], it was calculated that 100 replicate data sets were sufficient since initial simulation showed that the variance of the main parameter of interest, $\hat {E}_{s}$ is very low, together with high accuracy for moderate *θ* for the sigmoid random effects model introduced below. All fixed and varying parameters can be found in Table 1.

### Analysis of intervention effects in CRTs

#### Conventional linear analysis

The simplest analysis of a CRT of an intervention targeting mosquito densities and measuring malaria prevalence is a calculation of the risk ratio comparing prevalence in the two trial arms based on cluster level summaries. This leads to an estimate $\tilde {E}_{s}$ of effectiveness as

$$\tilde{E}_{s} = 1- \frac{\tilde{p}_{I}}{\tilde{p}_{C}}, $$

where $\tilde {p}_{C}$ is the proportion infected in the control arm and $\tilde {p}_{I}$ the proportion infected in the intervention arm. The more mosquito movement is introduced, the more the estimate $\tilde {E}_{s}$ is biased towards the mean between intervention and control arms as it does not adjust for the contamination.

Intervention estimates based on individual-level data and allowing for clustering can be obtained using generalized linear mixed effects models (GLMMs) with the trial arm as the dependent variable and a logistic link function. However, in the special case of binary data, fitting the logistic regression random effect models using Gaussian quadrature may not always provide an adequate model fit due to the failure of the numerical quadrature invoked. If this is the case, it is recommended in the literature to then fit the model with generalized estimating equations (GEEs) [30, p. 139; 31] and an exchangeable correlation structure [7, p. 220]. The estimated effectiveness $\tilde {E}_{s}$ is obtained as above, by comparing the model outputs $\tilde {p}_{C}$ and $\tilde {p}_{I}$. It is possible to extend these linear models with a term of the straight-line distance to the nearest discordant observation or a term of the density of households within a range that receive the intervention [4, 17, 18]. However, the contribution of each estimated coefficient remains linear and it is not possible to obtain a closed-form contamination range that specifies the maximal measurable extent of contamination from a linear model. It is also not possible to obtain this information from a model with a spatially structured random effect [4].

For malaria, interventions such as ITNs or indoor residual spraying are usually allocated to a household. The endpoint is then either measured in all residents of an area (as in the SolarMal trial [25]) or in a subgroup, normally children (as in the Navrongo trial [19]). If there is more than one observation per household, clustering within the household should also be allowed for in the analysis. If the trial outcome is malaria incidence instead of prevalence, the effectiveness can be calculated via a rate ratio including the time at risk for each group. Individual-level analysis can then use a logarithmic link function and an offset for the time at risk.

#### Proposal of a nonlinear analysis allowing for contamination

If an intervention lowers mosquito densities in intervention clusters, the intervention effects are contaminated between trial arms due to mosquito movement. This contamination depends on the distance of a household to the nearest discordant household and is expected to follow a symmetrical smooth gradient in the boundary area between intervention and control clusters. Let *Δ*_*ij*_ denote the distance of the *j*th household in the *i*th cluster to the nearest household in the other arm, endowed with a negative sign for the households in the control arm and a positive sign for households in the intervention arm (hereafter called nearest discordant household). This smooth gradient of intervention effectiveness across arms can then be modeled by a nonlinear sigmoidal function of *Δ*_*ij*_, governed by three parameters, *β*_1_ and *β*_2_, determining its position and height, and a parameter of steepness (growth rate) *β*_3_. A variety of functions can be used to model this sigmoidal shape, the most natural choice being the sigmoid or logistic function (hereafter called sigmoid model and abbreviated with $\mathcal {S}$):

$$\mathcal{S}(\Delta_{ij}) {\mathrel{\mathrel{\mathop:}=}} g^{-1}\Big(\beta_{1} + \frac{\beta_{2}} {1+\exp(-\beta_{3}\Delta_{ij})}\Big). $$

It is assumed that mosquito densities are proportional to the number of acquired infections, such that the analysis can be carried out with data on malaria prevalence. The function *g*^−1^ hence denotes a logit link function, adjusting $\mathcal {S}$ for binary outcome data. This model formulation can easily be extended for malaria incidence by using a log link function *g*^−1^ with a Poisson error function and an offset for the time at risk, and other functions than $\mathcal {S}$ are also possible.

**Fig. 2:**
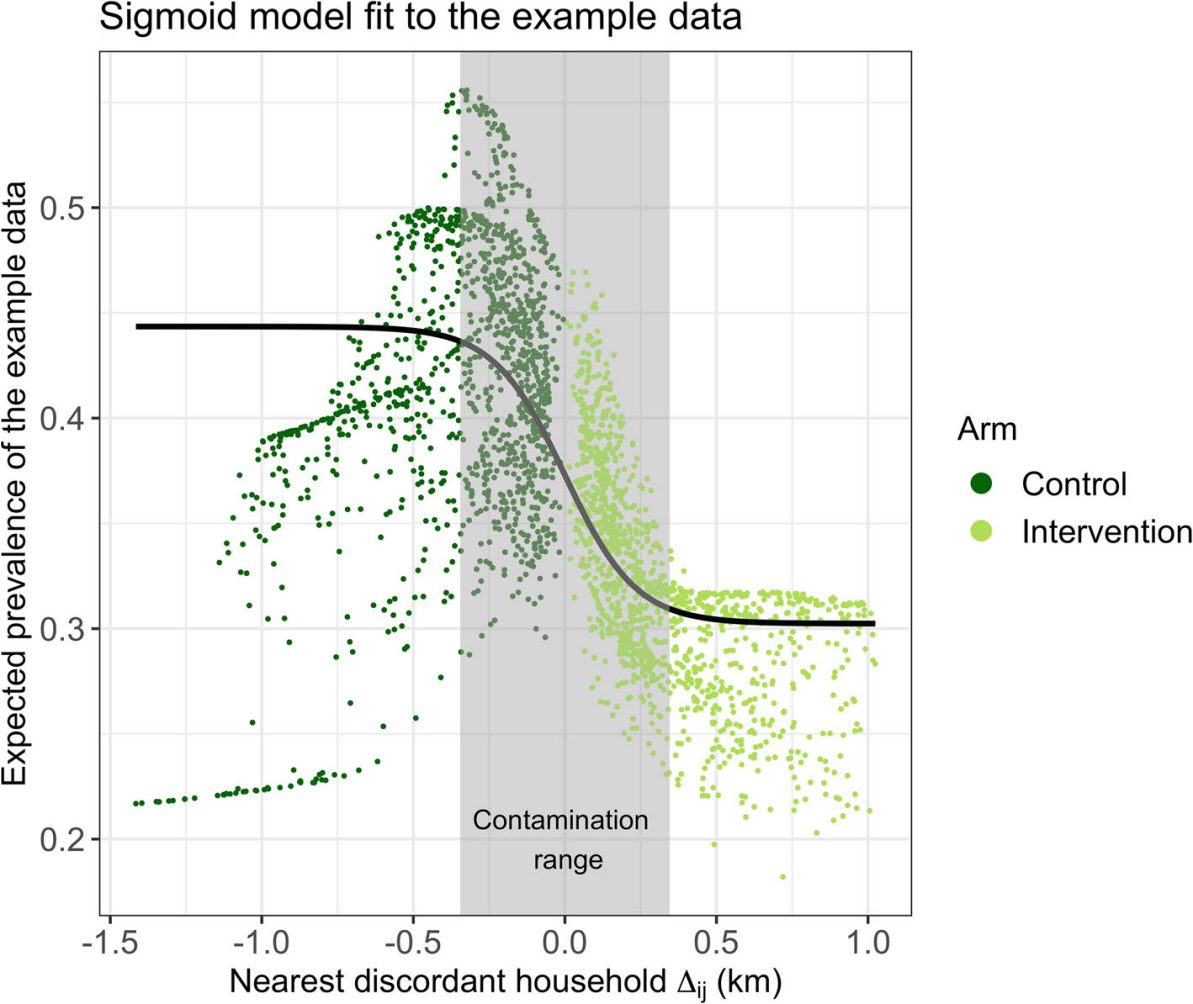
Illustration of the sigmoid model function for an example data set of the subsequently described simulation study. Households are arranged based on their distance to the nearest discordant household stratified by intervention status on the *x*-axis (*Δ*_*ij*_) and the expected prevalence is shown on the *y*-axis. The black line indicates the model fit and the gray rectangle the contamination range in both arms. The model is fitted to the same data set as is used in Fig. 1, the detailed parameters are listed there. The patterns in the expected prevalence (such as the approximately linear grouping in the control arm with low expected prevalence) arise from the location of households

The prevalences in the intervention and control arm are then defined as $\hat {p}_{C} = g^{-1}(\beta _{1})$, $\hat {p}_{I} = g^{-1}(\beta _{1} + \beta _{2})$ and the resulting effectiveness is $\hat {E}_{s} = 1 - \hat {p}_{I}/\hat {p}_{C}$. The parameter *β*_3_ can be transformed to a measure of contamination range in km, the distance over which the estimate of effectiveness is measurably biased. This is defined here as the value of *Δ*_*ij*_ where $\mathcal {S}$ attains 95% of its growth, i.e. $\mathcal {S}(\Delta _{ij}) = g^{-1}(\beta _{1} + 0.95\beta _{2})$. Solving this for *Δ*_*ij*_ results in an interpretable contamination range of $\hat {\theta } = \beta _{3}^{-1}\log (0.95/0.05) = 2.944\beta _{3}^{-1}$. An illustration of the sigmoid function as well as how it fits the expected prevalence of an example data set can be found in Fig. 2.

This sigmoid model can also be extended to allow for within-cluster correlation. One way to include a random effect for the clustering of the households is provided by Bayesian hierarchical models using Markov chain Monte Carlo (MCMC). A random effect *β*_1,*i*_ is assigned to each cluster, with the random effects centered on the expected prevalence in the control arm on a logit scale. For malaria prevalence, the outcome *Y*_*ij*_ of the *j*th household in the *i*th cluster can then be described as follows:

$$\begin{array}{*{20}l} Y_{ij} &\sim \operatorname{Binomial}(p_{ij}),\\ \operatorname{logit}(p_{ij}) &= \beta_{1,i} + \frac{\beta_{2}} {1+\exp(-\beta_{3}\Delta_{ij})},\\ \beta_{1,i} &\sim \operatorname{Normal}(\mu,\tau). \end{array} $$

Again, for malaria incidence, a log link function and an offset for the time at risk must be used. The other parameters *β*_2_, *β*_3_, *μ* and *τ* are assigned non-informative priors. Hereafter, this model will be called sigmoid random effects model, abbreviated with $\mathcal {S}_{RE}$.

Opposed to a conventional, linear analysis ignoring contamination, zones where contamination is likely have to be included in a sigmoid analysis. The precision and accuracy of the estimate of effectiveness $\hat {E}_{s}$ and the contamination range $\hat {\theta }$ not only depend on the size and number of clusters but also on the geographical size relative to the contamination range, the proximity of clusters in opposing arms and on the settlement distribution. This can be captured by considering the percentage of households unaffected by the contamination range $\hat {\theta }$ across the intervention boundary, namely the households whose distance to the discordant arm is greater than $\hat {\theta }$, hereafter called percentage of households in core, denoted by *ω*. To determine the premises under which a sigmoid analysis, either with $\mathcal {S}$ or $\mathcal {S}_{RE}$, yields precise and accurate estimates, the percentage of households in core *ω* will be used. A summary of all parameters introduced is listed in Table 2.

**Table 2:** Summary of the important parameters and abbreviations defined

$\tilde {E}_{s},\,\tilde {p}_{I},\,\tilde {p}_{C}$	Effectiveness and prevalences in the intervention and control arm (ignoring contamination)
*Δ*_*ij*_	Distance of *j*th household in the *i*th cluster to nearest discordant household
$\mathcal {S}$	Sigmoid model (allowing for contamination)
$\mathcal {S}_{RE}$	Sigmoid random effects model (allowing for contamination and including random effects)
*β*_1_, *β*_2_, *β*_3_	Parameters for $\mathcal {S}$ and $\mathcal {S}_{RE}$, describing the position, height and steepness of the function
$\hat {E}_{s},\,\hat {p}_{I},\,\hat {p}_{C}$	Effectiveness and prevalences in the intervention and control arm
	for $\mathcal {S}$ or $\mathcal {S}_{RE}$ (allowing for contamination)
$\hat {\theta }$	Estimated contamination range from $\mathcal {S}$ or $\mathcal {S}_{RE}$
*μ*, *τ*	Hyperparameters for the Bayesian hierarchical model $\mathcal {S}_{RE}$
*ω*	Percentage of households in core, that is households whose distance to the discordant arm is greater than $\hat {\theta }$ (ignoring assignment to arms)

#### Guide for the implementation in R

A trial can be analyzed with a sigmoid random effects model $\mathcal {S}_{RE}$ following the procedure outlined in Table 3. The R code as well as simulated datasets can be found in the additional files 1. As input, data on the trial is needed, along with some technical parameters to fit the MCMC model. The output is the estimated effectiveness and contamination range, with their 95% credible intervals (95%CI). In the first step, the distance to the nearest discordant household is calculated for each household, households in the control arm are additionally endowed with a minus sign. In the second step, the model is implemented as a Bayesian hierarchical model using MCMC, formulated in BUGS (Bayesian inference Using Gibbs Sampling) and fitted with JAGS [32] (Just Another Gibbs Sampler). The parameter *β*_3_ is constrained for the resulting contamination range to be interpretable, because $\hat {\theta }$ is calculated by taking the inverse of *β*_3_. The model is then fitted (third step) and the parameters are transformed (fourth step) according to the chosen link function, to be interpretable. The back transformation for *β*_3_ is independent of the link function, it holds that $\hat {\theta } = \log (0.95/0.05)\beta _{3}^{-1}$, as discussed above.

**Table 3:** Four steps to fit a sigmoid random effects model $\mathcal {S}_{RE}$ using MCMC

Input:	Geolocations of households; cluster and intervention assignment of households; trial outcome of interest (malaria prevalence or incidence); technical parameters for the MCMC
Output:	Estimated effectiveness and contamination range with 95%CI
1:	Calculate distance to the nearest discordant household
2:	Set up the sigmoid random effects model in JAGS
3:	Fit the sigmoid random effects model
4:	Transform the model output for interpretation

Both GEE and GLMMs are easily implemented in R with the packages geepack [33] and lme4 [34] for instance. The function $\mathcal {S}(\Delta _{ij})$ can be fitted to prevalence data at the household level with a maximum likelihood method for Bernoulli data, assuming that households are independent of each other. The optimization can then be performed with a genetic algorithm (GA package [35]).

### Analysis of simulations

Each data set was analyzed by the following: analysis allowing for within-cluster correlation (GEE), sigmoid model ($\mathcal {S}$), and sigmoid model including a random effect ($\mathcal {S}_{RE}$). For each of the 2×5×8 parameter configurations, the performance of the different models was assessed in terms of [28]: the relative bias with respect to the true value of the parameter of interest; the empirical standard error, that is the standard error of the parameter of interest; the average width of the 95% confidence intervals; and the coverage probability, the proportion of the 95%CI that contain the true value of the parameter of interest. The first two performance measures are on the parameter of interest itself, measuring its accuracy and precision across replicate data sets, the third and fourth are on the 95%CI around the parameter of interest, quantifying the precision and accuracy of the 95%CI. A summary with the corresponding formulae can be found in Table 4.

**Table 4:** Evaluation criteria for parameter estimations across replicate data sets. $\Theta \in \mathbb {R}$ denotes the true value of the parameter of interest, $\hat {\Theta } \in \mathbb {R}^{m}$ the parameter estimations for the *m* replicate data sets

Evaluation criteria	Abbreviation	Formula
Relative bias	relBias	$(\mathrm {E}[\hat {\Theta }] - \Theta)/\Theta $
Empirical standard error	EmpSE	$\text {Var}[\hat {\Theta }]^{1/2}$
Width of 95%CI	Width	Average width of 95%CI
Coverage probability	CP	Proportion of 95%CI that contained *Θ*

The 95%CI for the GEE and $\mathcal {S}$ analyses were calculated by parametric bootstrapping [36], because this method is very generalizable (R package boot [37]). This step was repeated *R*=100 times, leading to 10,000 resamples for each of the parameter configurations. For the fitting of $\mathcal {S}$, the parameter region for the genetic algorithm was chosen such that *β*_1_, *β*_2_∈[0,1] and *β*_3_>0. For the JAGS model, the 95% credible intervals were obtained from the 2.5 and 97.5 quantiles. Uninformative priors were chosen for *β*_1_ and *β*_2_ and a mildly informative prior for *β*_3_ to constrain the resulting contamination range to be in [0,1.5] km. All simulations were performed at sciCORE scientific computing core facility at the University of Basel under R version 3.6.0 [38].

## Results of the simulation study

The estimation of the two outcome parameters $\hat {E}_{s} \,=\, 1$$-\ \hat {p}_{I}/\hat {p}_{C}$ and $\hat {\theta }$ for the sigmoid models are evaluated by four performance measures (relBias, EmpSE, Width, CP) in terms of the four parameters that were varied (*E*_*s*_, *θ*, *c*, *h*) and compared against conventional methods for analysis. Both a GLMM and GEE showed very similar results and had acceptable model fit. However, a mixed effects model took slightly longer to fit. Simple cluster summaries also resulted in very similar results to a GEE or GLMM. Hence, only the results for a GEE analysis are used as comparison.This section is divided into three parts: first an evaluation of the simulations for the parameters *E*_*s*_ and *θ*, followed by the evaluation for the parameters determining cluster size and number of households per cluster, *c* and *h*. Each of these two parts is further divided based on the different performance measurements. The third part is on the results in terms of the percentage of households in core, *ω*, and the difference between a GEE and a sigmoid analysis.

### Varying the efficacy *E*_*s*_ and the contamination range *θ*

The results in this paragraph are averaged over all values of the number of clusters *c* and the number of households per cluster *h*.

#### Relative bias and empirical standard error

**Fig. 3:**
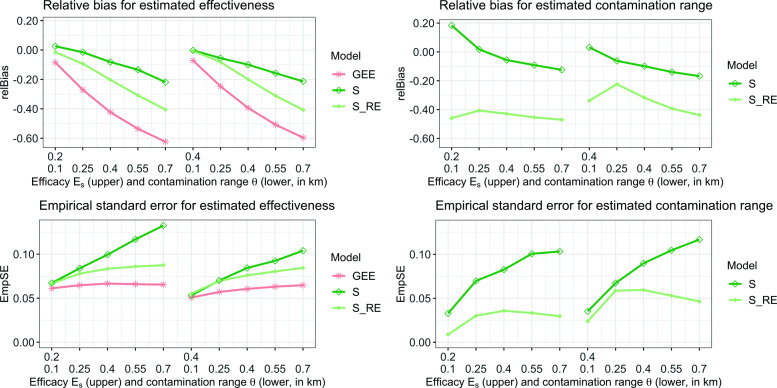
Relative bias (upper plots) and empirical standard error (lower plots) for model fits. The graphs on the left illustrate the errors for the parameter estimation $\hat {E}_{s}$ for each of the three models and the graphs on the right for the contamination range $\hat {\theta }$ that only exists for the sigmoid models $\mathcal {S}$ and $\mathcal {S}_{RE}$. Depicted on the *x*-axis are 10 different levels of parameter variation for *E*_*s*_ and *θ*

The relative bias and empirical standard error of the model fits are depicted in Fig. 3. As the assigned contamination range *θ* increases, the effectiveness estimate of the GEE is biased towards zero. Results are similar across different levels of assigned efficacy *E*_*s*_. The two sigmoid models also show greater bias towards zero with increasing contamination, but less so than with the GEE model. A similar pattern can be seen for the estimated contamination range $\hat {\theta }$ for $\mathcal {S}$, with greater bias towards zero as $\hat {\theta }$ increases. In contrast, the $\mathcal {S}_{RE}$ model estimates a constant contamination range regardless of the value of *θ*, but is always substantially more biased towards zero than for $\mathcal {S}$. The empirical standard errors show the exact opposite trends. For both parameter estimations, the empirical standard error increases with greater contamination. The GEE analysis has the lowest variance for the estimated effectiveness, and the $\mathcal {S}$ model the highest. In conclusion, a GEE analysis shows lower accuracy but higher precision than the sigmoid models, $\mathcal {S}$ shows high accuracy together with low precision and the $\mathcal {S}_{RE}$ is in between.

#### Width of 95%CI and coverage probability

The coverage probability and the width of the 95%CI are highly correlated: a desirable result would be a narrow 95%CI together with a high coverage probability. The results are depicted in Table 5. GEE always has narrow 95%CI but shows a very bad coverage probability for increasing *θ*. The sigmoid models have much better coverage probabilities but wider confidence intervals, with the random effect model yielding even wider confidence intervals, as was expected because $\mathcal {S}$ does not account for the clustering, leading to incorrectly high precision. For increasing *θ*, the width of the confidence intervals for the sigmoid models increases and the coverage probability decreases. This decrease in coverage probability is higher for a higher assigned efficacy *E*_*s*_. The width of the confidence intervals, however, is not altered by this parameter.

**Table 5:** Coverage probability and the width of the confidence intervals for the two parameters of interest, *E*_*s*_ and *θ* (10 levels)

		GEE		$\mathcal {S}$		$\mathcal {S}_{RE}$		
*E*_*s*_	*θ*	$\hat {E}_{s}$	$\hat {\theta }$	$\hat {E}_{s}$	$\hat {\theta }$	$\hat {E}_{s}$	$\hat {\theta }$	
0.2	0.1	82	–	85	100	96	100	CP (%)
		0.18	–	0.20	0.30	0.27	0.30	Width
	0.25	70	–	88	100	94	100	
		0.18	–	0.27	0.62	0.30	0.77	
	0.4	52	–	91	100	93	100	
		0.19	–	0.33	0.95	0.33	1.19	
	0.55	42	–	90	100	88	100	
		0.19	–	0.39	1.27	0.34	1.59	
	0.7	34	–	90	100	84	100	
		0.20	–	0.46	1.61	0.35	1.99	
0.4	0.1	80	–	88	100	96	100	
		0.15	–	0.18	0.22	0.23	0.30	
	0.25	37	–	90	100	94	100	
		0.17	–	0.25	0.45	0.29	0.78	
	0.4	11	–	90	99	85	100	
		0.18	–	0.30	0.70	0.32	1.18	
	0.55	2	–	92	100	71	100	
		0.19	–	0.36	0.98	0.33	1.54	
	0.7	0	–	91	99	54	100	
		0.20	–	0.42	1.29	0.35	1.91	

### Varying the number of clusters *c* and the number of households in each cluster *h*

The results in this paragraph are averaged over all values of efficacy *E*_*s*_ and contamination range *θ*.

#### Relative bias and empirical standard error

**Fig. 4:**
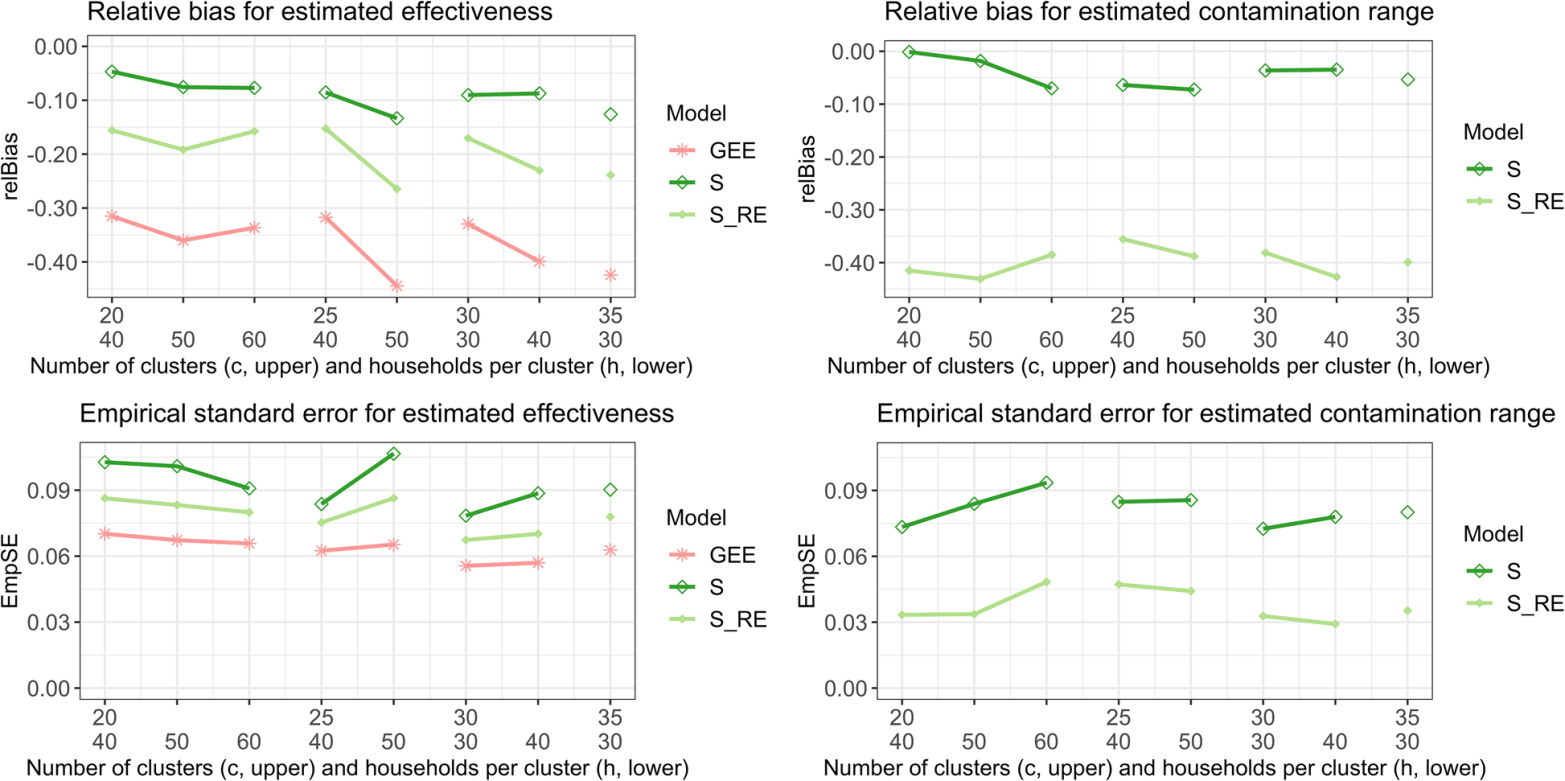
Relative bias (upper plots) and empirical standard error (lower plots) for models fits when the parameters *c* (number of clusters in one arm, upper *x*-axis) and *h* (households per cluster, lower *x*-axis) are varied. The graphs on the left illustrate these errors for the parameter estimation $\hat {E}_{s}$ and the graphs on the right for $\hat {\theta }$ (only for sigmoid models)

The relative bias and empirical standard error of the model fits are visualized in Fig. 4. All three models are quite robust with respect to the different variations of *c* and *h*. Again, as noted above, a GEE has the lowest accuracy and highest precision, while $\mathcal {S}$ shows the opposite. For all models, the empirical standard error is almost constant for both estimated parameters.

#### Width of 95%CI and coverage probability

The width of the confidence intervals and the coverage probability yield no new insights; the results are depicted in Table 6. Again, the sigmoid models have a very high coverage probability whereas this is very low in GEE. $\mathcal {S}_{RE}$ has wider credible intervals than $\mathcal {S}$, with comparable coverage probability.

**Table 6:** Coverage probability and the width of the confidence intervals for the two parameters of interest, *c* and *h* (8 levels)

		GEE		$\mathcal {S}$		$\mathcal {S}_{RE}$		
*c*	*h*	$\hat {E}_{s}$	$\hat {\theta }$	$\hat {E}_{s}$	$\hat {\theta }$	$\hat {E}_{s}$	$\hat {\theta }$	
20	40	59	–	89	100	91	100	CP (%)
		0.20	–	0.33	0.79	0.34	1.01	Width
	50	48	–	87	100	87	100	
		0.19	–	0.33	0.92	0.33	1.21	
	60	40	–	88	100	88	100	
		0.18	–	0.29	0.81	0.31	1.17	
25	40	41	–	86	99	88	100	
		0.17	–	0.27	0.73	0.30	1.11	
	50	30	–	86	100	81	100	
		0.19	–	0.35	0.95	0.32	1.30	
30	30	48	–	91	100	93	100	
		0.16	–	0.27	0.75	0.28	1.01	
	40	47	–	93	100	86	100	
		0.19	–	0.35	0.90	0.31	1.19	
35	30	32	–	90	100	80	100	
		0.19	–	0.33	0.83	0.31	1.16	

### Comparison between the analyses based on the percentage of households in core

**Fig. 5:**
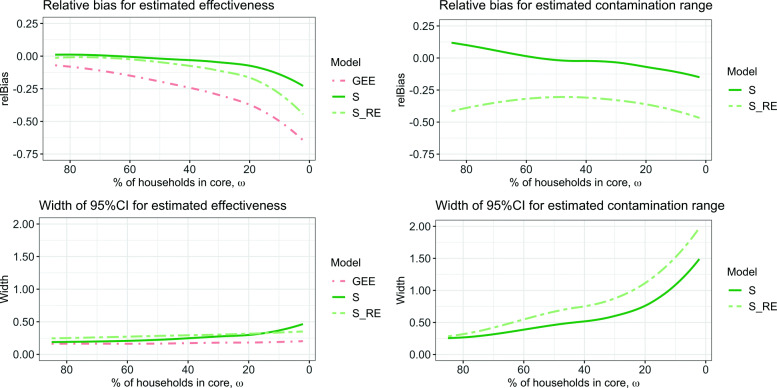
Relative bias (upper plots) and width of the 95%CI (lower plots) for model fits in terms of the percentage of households in core *ω*. The graphs on the left illustrate these errors for the parameter estimation $\hat {E}_{s}$ and the graphs on the right for $\hat {\theta }$. The lines are smoothed over all parameters *E*_*s*_, *θ*, *c* and *h*

The percentage of households in core, *ω*, is the key indicator for determining how small clusters can be with respect to the contamination range $\hat {\theta }$ for the sigmoid models. This measurement does not differentiate between households in the intervention or control arm, but if an equal number of same size clusters are allocated to both arms, it is likely that there is a certain balance. For each of the simulations, *ω* was calculated, and the relative bias together with the width of the 95%CI was plotted with respect to *ω*; see Fig. 5. This figure displays the same data as described in Figs. 3 and 4, but with respect to *ω*. From the graphs displaying the relative bias and width of the 95%CI for the estimated effectiveness $\hat {E}_{s}$, it becomes clear that at ≈20*%* of households in core, the dynamics of the curves change. In all three models, there is considerably more bias moving towards less households in core. For a GEE analysis, it is clearly beneficial to have *ω*=100*%*, all households in core. For a sigmoid analysis (with $\mathcal {S}$ or $\mathcal {S}_{RE}$), this is not the case. For the estimated effectiveness, if around 50% of the households are in core, the relative bias is approximately zero and the width of the confidence intervals is still small compared to the width if fewer households were in core. The relative bias for the estimated contamination range $\hat {\theta }$ is very flat and shows a nonlinear behavior. The width of the 95%CI increases the fewer households there are in core, and once less than *ω*≈20*%*, the growth accelerates substantially.

## Example: the Navrongo trial of ITNs

### Study design

This large-scale CRT was conducted between July 1993 and June 1995 in the Kassena-Nankana districts of northern Ghana with the goal to assess the effect of ITNs compared to no ITNs on child mortality. The area was predominantly rural with people living in dispersed settlements, arranged in compounds. The study was a parallel CRT with 96 geographically contiguous clusters and an average of 120 compounds per cluster. Where possible, small paths or roads were used to delineate the clusters, but in most cases, the cluster boundaries did not correspond to natural barriers. The intervention of permethrin impregnated bed nets was allocated to 48 randomly chosen clusters and 31,000 ITNs were provided to intervention participants. A full description of the study design is reported elsewhere [19].

The outcome was all-cause mortality in children aged 6 months to 4 years, reported as a standardized mortality ratio (SMR). All children in the study area were included. The expected number of deaths for each cluster was computed by applying age-specific death rates derived from the pre-intervention population to the post-intervention time at risk and was treated as an offset for the regression models [16]. Data captured included the geographical coordinates of the household and the distance from each household to the nearest discordant household.

### Published trial results

A total of 857 deaths occurred among children in the trial over the 2 years of follow-up. The original analysis found a 17% reduction in mortality (rate ratio (RR) comparing SMRs of 0.83, 95%CI [0.69,1.00]) [19]. Subsequently, Binka et al. [16] graphed the ITN effect in relation to distance from the boundary. A regression approach incorporating this distance indicated that among children from clusters randomized to the control arm, the mortality risk increased by 6.7*%* with each additional shift of 100 m away from the nearest household in the intervention arm (95%CI [1.8,11.4]*%*) [16]. Notably, due to the considerable spatial information available, the estimated confidence intervals (which did not allow for the spatial auto-correlation in the data) around the regression lines in this analysis were narrow, even though the overall estimate of effectiveness was imprecise [19]. This data set was recently reanalyzed [39] using multilevel models and geostatistical approaches to allow for spatial correlations and contamination effects. Including the distance to the nearest discordant household as a fixed effect in the multilevel model indicated an increase of the SMR with every additional 100 m away from the intervention arm of 1.7*%* (95% credible interval [0.6,2.6]*%*) [39]. The main conclusion of the reanalysis was that, despite the evidence of a spatial contamination effect, the primary conclusions of the trial remain unaffected. The increase of the SMR with every additional 100 m was estimated to be less than was reported before, but the confidence intervals were similarly narrow. The confidence intervals around the main effect remained wide.

### Methods

The Navrongo data was reanalyzed with the sigmoid models $\mathcal {S}$ and $\mathcal {S}_{RE}$ and the results were reported in terms of mortality incidence rates. Hence, a log link function *g*^−1^ with a Poisson error function was used. As in the original analysis, the expected number of deaths was treated as an offset. For comparison with the original spatial analysis, the increase in mortality rate with each additional 100 m away from the boundary was calculated by comparing the SMRs at the required distances. For fitting the JAGS models, the number of iterations was set at 20,000 with a burn-in period of 10,000 and uninformative priors were used for *β*_1_ and *β*_2_, together with a mildly informative prior for *β*_3_, as for the simulation study. For fitting the sigmoid models without a random effect, *R* was set to 1000, and the valid parameter region for the search of the genetic algorithm for the contamination range was chosen to be [0.05,0.6] km. An extended reanalysis of the Navrongo data can be found in the appendix.

### Results

**Fig. 6:**
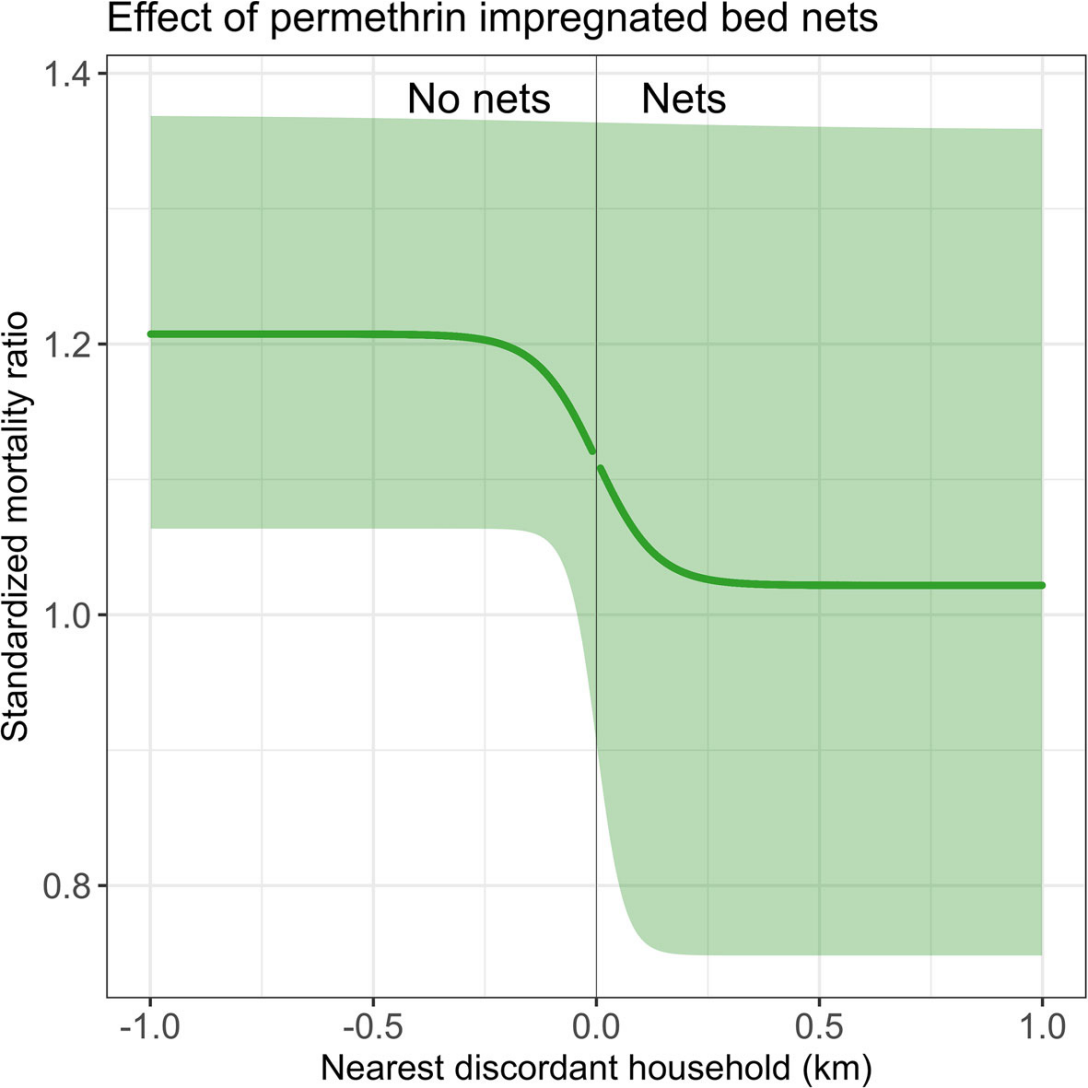
Illustration of the results for the sigmoid model $\mathcal {S}_{RE}$. The thick line indicates the fitted sigmoid curve, together with the confidence intervals. On the *x*-axis, the distance to the nearest discordant household up to 1 km and on the *y*-axis, the standardized mortality ratio is plotted

Bed nets were associated with a 16.6*%* and 19.0*%* reduction in all-cause mortality in children aged 6 months to 4 years for $\mathcal {S}_{RE}$ and $\mathcal {S}$, the sigmoid models with and without a random effect, respectively. As in the original analysis, confidence intervals were wide. Contamination across the boundary was found to be around 0.2 km per arm, again with wide confidence intervals, especially for $\mathcal {S}_{RE}$. The parameter estimations for $\mathcal {S}_{RE}$ translate to an increase in mortality from the intervention boundary of 5.6*%*, 95%CI [0.2,15.5]*%* up to 100 m and 2.4*%*, 95%CI [0.1,1.4]*%* between 100 m and 200 m. After that, the increase is very slow, since the contamination range is around 200 m. Since the model is symmetrical, the same numbers also hold for a decrease in mortality with each 100 m away from the nearest household without a bed net. All the estimates are displayed in Table 7. Figure 6 illustrates the results for $\mathcal {S}_{RE}$, analogous to Fig. 2.

**Table 7:** Results for the parameter estimations for the Navrongo data for sigmoid models $\mathcal {S}$ and $\mathcal {S}_{RE}$, compared to the results of the original analysis [16] and a previous reanalysis [39]. In brackets, the 95%CI are given. The contamination range only exists for $\mathcal {S}$ and $\mathcal {S}_{RE}$. The last column indicates the increase in mortality after 100 m away from the nearest discordant household. For $\mathcal {S}_{RE}$ this is nonlinear, hence the increase after 100 m and 200 m is reported

	Effectiveness	Contamination range	Increase mortality shift 100m; 200m
$\mathcal {S}_{RE}$	16.6*%*, [2.2, 30.7]*%*	0.198 km, [0.092, 1.088] km	5.6*%*, [0.2,15.5]*%*; 2.4*%*,[0.1,1.4]*%*
$\mathcal {S}$	19.0*%*, [7.7, 28.1]*%*	0.170 km, [0.051, 0.495] km	–
Original [16]	17.0*%*, [0.0, 31.0]*%*	-	6.7*%*, [1.8,11.4]*%*
Reanalysis [39]	18.0*%*, [5.0, 30.0]*%*	-	1.7*%*, [0.6,2.6]*%*

## Discussion

When contamination is anticipated in a cluster randomized trial of a malaria control intervention, a conventional analysis would lead to a biased estimate of effectiveness. To avoid this, a fried egg design is often recommended, attempting to separate the trial arms with buffer zones around each cluster [7]. This allows a conventional analysis, for instance with GEEs or GLMM, to be carried out, but leads to trials of much bigger geographical size than would be needed based on sample size formulae [26, 27]. Further, an estimate of the measurable contamination range is needed to quantify the buffer zone, and in the absence of suitable data this is typically based only on expert opinion. The contamination between arms in a cluster randomized trial contains information about the intervention per se. An analysis that takes this information into account as a trial outcome can lead to unbiased estimates of effectiveness, even when a substantial part of the data is affected by this contamination. This work proposes such an analysis for CRTs where contamination of intervention effects is introduced by mosquito movement and a nonlinear model is used to quantify the range of contamination across intervention arms.

The main strength of this approach lie in the adjustment of the estimate of intervention effectiveness to account for contamination and yielding a closed-form estimate for the contamination range that can inform future analyses. Obtaining such a simple closed-form estimate would not be possible from a linear parametric or a nonparametric approach. The sigmoidal shape functions make the model nonlinear, complicating the analysis substantially. This also implies that an interpretation of the coefficients is not as straightforward as for a linear model and the contamination range needs a back transformation. Furthermore, the sigmoid function is symmetrical, which means that if there was an asymmetrical contamination, such as a protective effect of an intervention on nearby non-users, but no increase in risk for the intervention users associated with being near the boundary, this would not be captured with this proposed analysis. To capture this, an asymmetric function with another parameter for the asymmetry would be needed, further complicating the analysis and interpretation.

The fitting of the random effects model was performed with an MCMC approach, where the binary structure of the outcome, representing a malaria prevalence survey, can easily be treated. The inclusion of random effects in a frequentist approach [40] with the R package nlme [41] proved unreliable, because nlme does not allow for binary outcome data structure. Representing the common practice in the field, an exchangeable correlation structure was chosen. For the Navrongo trial, a recent reanalysis [39] tested the impact of different spatial correlations and found minimal differences, supporting the choice of an exchangeable correlation structure.

The simulation study indicated that different cluster configurations (number of clusters and number of households per cluster) only slightly influenced the performance. This makes sense because rearrangement of households based on their distance to the intervention boundary does not take account of cluster assignment; the overall number of households and their spatial distribution relative to the boundary is more important. The results were not very sensitive to the assigned level of efficacy and a low efficacy of 20% did not impose fitting problems. Mosquito movement and hence contamination was simulated with varying widths of normal kernels centered at the households. An increase in mosquito movement biased the intervention effects towards the null in all of the analyses, but the bias was less extreme for the sigmoid functions.

It would be possible to extend the GEE model used for comparison with a term of the straight-line distance to the nearest discordant observation [4] and to include contamination as a parameter that quantifies the increase in effectiveness per distance unit away from the intervention boundary, as it has been done in the original analysis of the Navrongo trial [16]. In an initial analysis of the simulation study using this approach, it was found that the main parameter of effectiveness was not affected (results not shown). Hence, only the more basic GEE model is used for comparison to keep the focus of the simulation study on the sigmoid models.

The results of the simulation study were obtained by averaging over scenarios and varying parameters. This should be kept in mind when interpreting these results. Also, the variation arising due to a finite number of simulations that could be assessed by Monte Carlo standard errors [29] was not addressed. More work is needed to better understand the differences between linear models incorporating contamination and nonlinear approaches and to better determine the premises under which a sigmoid model is suitable.

Since the performance of a sigmoid analysis, apart from the parameters that were varied in the simulation study, also depends on other factors such as geographical cluster size, we explored the simulations with respect to the percentage of households in core, i.e., households that are unaffected by the contamination range across the boundaries, which is a scale-free parameter. Usually, parameter configurations with a similar ratio between cluster size h and contamination range led to similar values of the percentage of households in core. When more than ≈50*%* of the households are in core, the simulations indicated that it is possible to estimate the effectiveness without bias, irrespective of the cluster division.

Since $\mathcal {S}_{RE}$ adjusts for the clustering, it is certainly to be preferred for primary analyses of efficacy over $\mathcal {S}$, although $\mathcal {S}$ yields better results for estimating the contamination range. Hence, these models are not a panacea for contamination in CRTs. As validation, the information gained from both models could be used to define buffer zones post hoc. A range of different buffer zones could be used, and the resulting estimates of effectiveness could be compared to the sigmoid model to check how the estimated contamination range relates to the size of buffer needed to avoid bias. Furthermore, it would be desirable to assemble estimates from multiple previous field studies, before an appropriate value of the contamination range can be assumed for use in designing a new trial for any specific site.

This analysis raises the question of how best to divide populations into clusters. Many CRTs are designed with individual villages as clusters, which, depending on the settlement pattern, generally achieves spatial separation of trial arms by ensuring that cluster boundaries pass through unpopulated areas between villages. However, this approach leads to heterogeneity between clusters and varying cluster size (though a uniform number of households might be sampled in each cluster). If estimation of the contamination function is considered desirable, it may be important for some cluster boundaries to pass through inhabited areas rather than avoiding them. This makes it feasible to define clusters with equal numbers of enrolled individuals, as was done in the simulation study. It is attractive to use an algorithmic approach to cluster assignment in a CRT, for instance using a travelling salesman algorithm [24, 25] as we did in the simulations. Further analysis would be needed to determine whether this is optimal in terms of maximizing trial efficiency.

A reanalysis of the Navrongo trial of the effect of ITNs on child mortality in northern Ghana with the proposed method yielded similar results to the original analysis [19]. For the sigmoid model $\mathcal {S}_{RE}$, bed nets were associated with a 16.6*%* reduction in all-cause mortality in children aged 6 months to 4 years (95%CI [2.2, 30.7]*%*) with a contamination range of 0.198 km per arm (95%CI [0.092, 1.088] km). Given that the outcome was all-cause mortality in children aged 6 months to 4 years and hence the data are rather sparse, it is unsurprising that the credible intervals for both the effectiveness and the contamination range estimate are wide. The result for the contamination is in line with what was found in a larger trial of ITNs in Asembo, Kenya [18], where significant protective effects of ITNs were found for distances of up to 300 m from cluster boundaries. In the original spatial analysis of the Navrongo data [16], an increase with each 100 m away from the nearest household with a bed net was reported to be 6.7*%* (and 1.7*%* for the spatial reanalysis, both with narrow confidence intervals). Since the sigmoid model $\mathcal {S}_{RE}$ is nonlinear, the increase with each unit is not a constant. The findings here of 5.6*%* increase in mortality for the first 100 meters and then 2.4*%* increase from 100−200 m are similar to the previously reported results of 6.7*%*. The Navrongo trial had very large clusters and many households were unaffected by the estimated contamination range. It can be seen in this example that even when the contamination range is big enough to be estimable with such methods, this need not make much difference to the estimate of effectiveness.

An extension of the analyses in this paper would be to build on the results on the percentage of households in core by transforming the distance to the boundary into a measure of local coverage of the intervention and hence estimating the effectiveness as a function of coverage. This framework could also easily be extended to account for another hierarchy of clustering at the household level or to trial designs with repeated sampling of individuals for either incidence or prevalence, using random effects terms to account for individual variation in addition to cluster effects. More estimates of the contamination range from other field studies are needed to design further trials. Guidelines for how to design CRTs for such an analysis as well as reanalyzes of other CRTs are planned.

## Conclusions

Contamination measures are themselves valuable trial outcomes, providing information about the indirect effects of the intervention, and calculation of quantities derived from them might have several motivations. For some interventions, such as those intended to repel mosquitoes, the extent of contamination directly relates to the action of the intervention and will inform the density at which deployment is required. For any intervention, demonstration of significant contamination confirms that there is effectiveness: it is not possible for contamination to occur unless the two arms of the trial differ in the outcome. Estimates of this contamination range could be used to define buffer zones post hoc (using pre-specified criteria). But–more importantly–the possibility of statistically adjusting for contamination suggests not only that the size of buffer zones could be minimized, but that they could be completely avoided, leading to smaller and more cost-efficient trials of malaria interventions.

## Appendix

### Extension of the reanalysis for the Navrongo trial

The previous reanalysis [39] indicated that adjusting the main outcome of mortality for a contamination effect did not influence the results. At the same time, the confidence intervals around the contamination effect were confirmed to be narrow. It is hence assumed that clusters were chosen to be so large that even a contamination range of several hundred meters did not affect the main outcome, i.e., the percentage of households in core was very high. As shown above, for a sigmoid random effects analysis ($\mathcal {S}_{RE}$) to result in precise and accurate estimates of effectiveness, only around 50% of households need be in core. The trial cannot be redone with smaller clusters (and hence ≈50*%* of households in core), but simulations can show whether the estimate of effectiveness remains stable for a trial with smaller clusters. For each cluster, a subset of households far away from the discordant trial arm can be randomly excluded. This reduces the number of households per cluster without violating the cluster boundaries. Because only households far away from the discordant trial arm are chosen for exclusion, the percentage of households, *ω*, decreases. This could be seen as the opposite of a fried egg design, since in each cluster, households close to a discordant household are kept. It is hypothesized that a GEE analysis is more biased the more households in core are excluded and a sigmoid model analysis remains unaffected because the information of the contamination range remains the same, although confidence intervals will probably get wider.

If only households in core were eligible for exclusion, the resulting cluster sizes would be imbalanced, since the number of households in core in each cluster varies significantly. Hence, households lying further away from the nearest discordant household than the 20% quantile were eligible for exclusion. Of these 80% of the households in each cluster, a percentage *q* was randomly selected and 0.8*q* households were randomly excluded. In total, 50 values for *q* were chosen and for each of those values, 50 replicate data sets were generated. The estimates were bootstrap corrected for 100 resamples, for the JAGS model the number of iterations was set to 2000 with a burn-in period of 500.

**Fig. 7:**
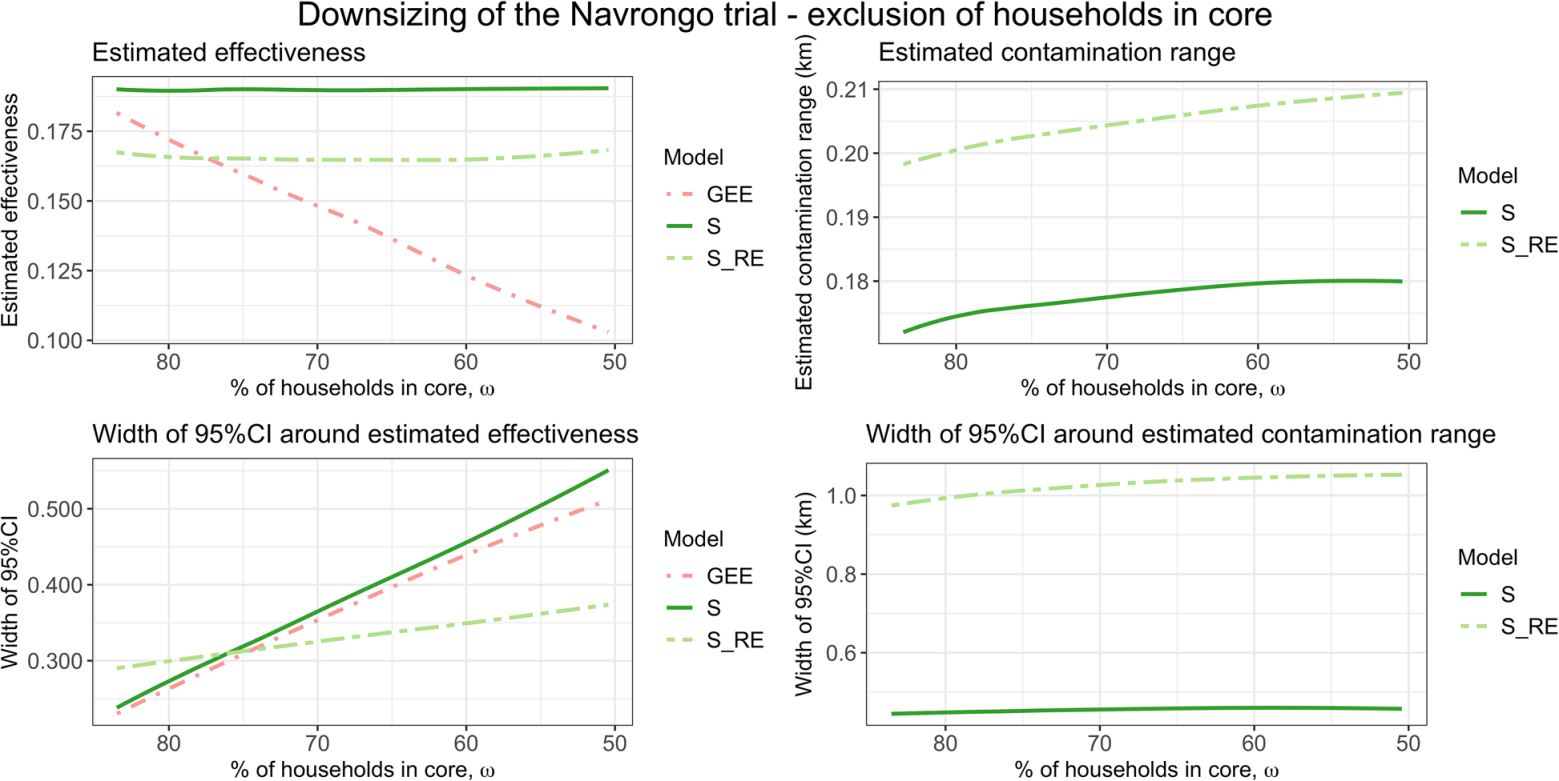
Reanalysis of the Navrongo trial, where for each cluster, households far away from the intervention boundary were randomly excluded to vary the percentage of households in core *ω*. The estimated parameters (upper plots) and width of the 95%CI (lower plots) for a GEE, $\mathcal {S}$ and $\mathcal {S}_{RE}$ analysis in terms of the percentage of households in core are visualized

For a contamination range of 0.198 km, 82% of the households were in core and hence unaffected by the estimated contamination range. If only 50% of the households had been in core, the results for the sigmoid models remain unaffected, but with slightly wider confidence intervals. The result is displayed in Fig. 7. For the GEE model, the estimated effectiveness decreases the more households are excluded (and hence the fewer households are in core) as expected. The estimated effectiveness for the sigmoid models $\mathcal {S}_{RE}$ and $\mathcal {S}$ remains constant, even when only ≈50*%* of households lay in core, corresponding to an exclusion of 64% of all households in the trial. The width of the 95%CI around the estimated effectiveness increases linearly for all three models, because fewer households (and hence smaller clusters) leads to a loss of power. This increase is slower for $\mathcal {S}_{RE}$. The estimated contamination range remains in the magnitude of 200 m, a slight increase for both models is noted as *ω* decreases. The width of the 95%CI for the estimated contamination range is constant for different *ω* (but quite large for $\mathcal {S}_{RE}$, around 1 km).

The conclusion that the Navrongo trial could have been much smaller without the results being affected hence holds. With only 36% of the original households included, the same parameter estimations are attained with a sigmoid random effects model, although the width of credible intervals increases. This underlines the findings from the simulation study that clusters could be much smaller in terms of number of households included and information from households close to the boundary should not be discarded.

## Supplementary Information

**Additional file 1** Analysis_CRT.R. R code for a sigmoid random effects model for the analysis of a CRT of malaria prevalence.

**Additional file 2** simulated_trial.RData. Simulated data file for the R code in Analysis_CRT.R.

## Data Availability

An example of the simulated data is available in the additional files 2.

## Abbreviations

CRTs: Cluster randomized trials
ITN: Insecticide-treated net
RDT: Rapid diagnostic test
TSP: Traveling salesman problem
ICC: Intra-cluster correlation coefficient
GLMM: Generalized linear mixed effects model
GEE: Generalized estimating equations
MCMC: Markov chain Monte Carlo
BUGS: Bayesian inference using Gibbs sampling
JAGS: Just another Gibbs sampler
GA: Genetic algorithm
relBias: Relative bias
EmpSE: Empirical standard error
CP: Coverage probability
SMR: Standardized mortality ratio
RR: Rate ratio
